# Expression of a Chlorophyll *b* Reductase Gene from *Zoysia japonica* Causes Changes in Leaf Color and Chlorophyll Morphology in *Agrostis stolonifera*

**DOI:** 10.3390/ijms23116032

**Published:** 2022-05-27

**Authors:** Di Dong, Zhuoxiong Yang, Yuan Ma, Shuwen Li, Mengdi Wang, Yinruizhi Li, Zhuocheng Liu, Liebao Han, Yuehui Chao

**Affiliations:** School of Grassland Science, Beijing Forestry University, Beijing 100083, China; didos12@bjfu.edu.cn (D.D.); yangzhuoxiong@bjfu.edu.cn (Z.Y.); ma_yuan0@163.com (Y.M.); lishuwen@bjfu.edu.cn (S.L.); mengdi0627@163.com (M.W.); liyinruizhi@bjfu.edu.cn (Y.L.); liuzhuocheng@bjfu.edu.cn (Z.L.)

**Keywords:** ZjNOL, NYC-like, creeping bentgrass, chlorophyll *b* reductase

## Abstract

The NYC-like (NOL) enzyme is considered as an essential enzyme for chlorophyll *b* degradation, which catalyzes the formation of 7-hydroxymethyl chlorophyll a from chlorophyll *b*. The *ZjNOL* gene was cloned from *Zoysia japonica* with a completed coding sequence of 981-bp in length, encoding 326 amino acids. ZjNOL was localized on the stroma side of the thylakoid membrane, and co-localized with ZjNYC in the chloroplasts. Multiple photoregulatory elements and hormone regulatory elements were identified in the promoter region of the ZjNOL gene, and the expression level of the *ZjNOL* gene was dramatically up-regulated in senescence leaves, which were regulated by a variety of plant hormones. *ZjNOL*’s ectopic expression in creeping bentgrass produced yellow leaves, thicker cortex, and smaller vascular column cells. Additionally, transgenic plants exhibited morphological alterations in their chloroplast structure, and the number of grana and thylakoids per grana stack reduced dramatically. Transgenic plants also had a lower photosynthetic rate and Fm/Fv than the control. The transgenic plants displayed a decreased chlorophyll content and a greater rate of ion leakage. The properties and activities of *ZjNOL* will serve as a foundation for future research into gene functions and regulatory processes.

## 1. Introduction

**Figure 1:**
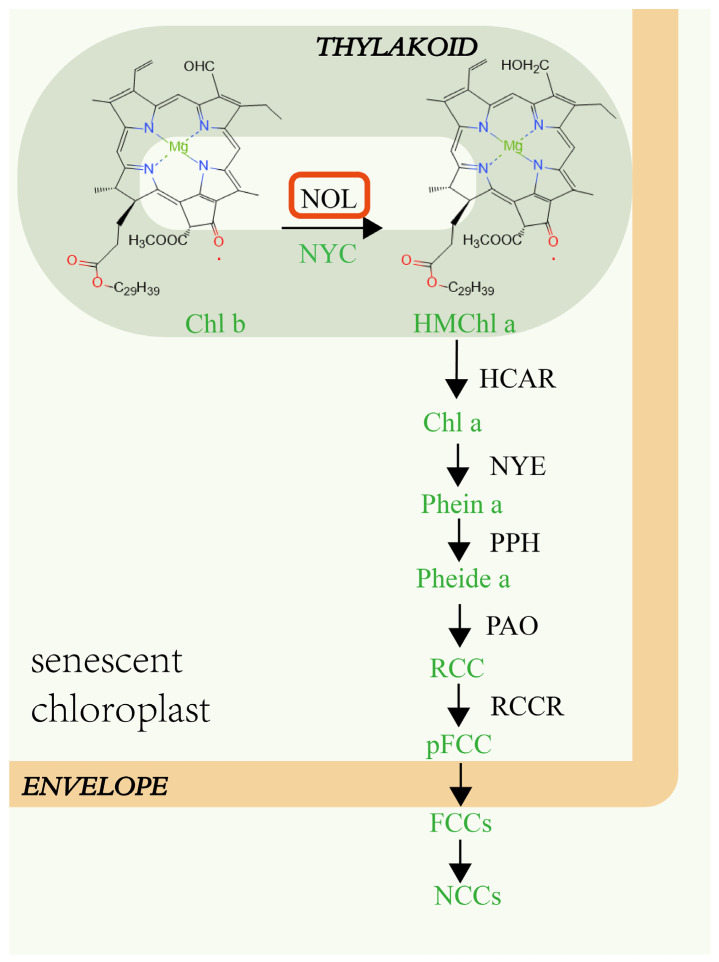
NOL is involved in regulating chlorophyll degradation in a senescing mesophyll cell of Arabidopsis thaliana [5]. HMChl a, 7-hydroxymethyl chlorophyll a; HCAR, chlorophyll a reductase; NYE, non-yellowing 1; PPH, pheophytinase; PAO, pheophorbide a oxygenase; RCCR, red chlorophyll catabolite reductase; RCC, red chlorophyll catabolite; pFCC, primary blue-fluorescent chlorophyll catabolite; FCCs, fluorescent chlorophyll catabolites; NCCs, nonfluorescent chlorophyll catabolites; Pheide a, pheophorbide a; Phein a, Pheophytin a.

Leaf senescence is a dynamic process in which plants recover nutrients from senescent leaves. Leaf yellowing is a clear indicator of leaf senescence, due to chlorophyll breakdown and carotenoid color exposure. During senescence, cells must degrade free chlorophyll and its color-decomposed metabolites quickly in order to escape photooxidation toxicity [1]. In higher plants, chlorophyll is a component of the polysubunit thylakoid membrane protein complex, and it occurs in two forms, chlorophyll *a* (*Chl a*) and chlorophyll *b* (*Chl b*). *Chl a* is found in all chlorophyll protein complexes, but *Chl b* is found only in the light-harvesting complex I and II (LHCI and LHCII), which make up the photosystem I complex and photosystem II complex, respectively [2]. LHCII is mostly found in the grana in the thylakoid membrane’s accumulation area, where it plays a critical role in the creation and maintenance of grana accumulation [3]. Chlorophyll degradation enzymes (CCEs) have been found to interact with photosynthetic organs. During leaf senescence, LHCII can interact with five chlorophyll-degrading enzymes (NYC, NOL, PPH, PAO, and RCCR) [4]. Chlorophyll *b* is converted to chlorophyll a via a two-step process involving chlorophyll *b* reductase and 7-hydroxymethyl chlorophyll a reductase (HCAR) (Figure 1) [5]. NYC (non-yellow coloring) and NOL (NYC1-like) are two distinct subtypes of chlorophyll *b* reductase [6]. The end product of chlorophyll degradation is a colorless water-soluble linear tetrapyrrole referred to as nonfluorescent chlorophyll catabolites (NCCs), which are stored in the vacuoles of senescent cells. The NOL gene controls the initial degradation of *Chl b* and promotes its transition into *Chl a* [7]. All non-fluorescent chlorophyll breakdown metabolites in higher plants are produced from *Chl a*, indicating that the transformation of *Chl b*, mediated by chlorophyll *b* reductase, is the first step and a critical link in chlorophyll degradation [8].

In *Arabidopsis thaliana*, overexpressed *NOL* plants had a considerably lower chlorophyll content than wild-type plants, and photosystem II had a reduced antenna size, indicating that *NOL* was engaged in photosystem II antenna size control [9]. A phylogenetic study of the rice (*Oryza sativa*) *OsNOL* gene revealed that the closest protein to *OsNOL* was *OsNYC1*. Rice *nol* mutants exhibited a phenotype similar to that of the *nyc1* mutant, with significant suppression of chlorophyll *b* degradation. Additionally, LHCII was preserved selectively throughout senescence and thylakoid grana were retained even at a late stage of senescence [10]. The *LpNOL* RNA interference (*NOLi*) in perennial ryegrass (*Lolium perenne*) dramatically suppressed chlorophyll breakdown during leaf senescence and altered the leaf color of perennial ryegrass, implying that NOL functions as a chlorophyll *b* reductase and is also associated with the perennial herb’s middle color phenotype [11]. 

*Zoysia japonica* is a typical warm-season turfgrass found around the world. Due to its drought and salt resistance, it is commonly utilized on golf courses, sports fields, and urban greening. Creeping bentgrass (*Agrostis stolonifera*) is a significant cool-season grass species that is extensively used on golf courses as turfgrass [12]. While the genes involved in chlorophyll breakdown have been identified, their regulatory roles in plant growth and development remain unknown. In this study, the *ZjNOL* gene from *Zoysia japonica* was isolated and identified, and its ectopic expression and functional studies in creeping bentgrass were carried out to gain a better understanding of the *NOL* gene’s function and to lay the groundwork for future research on its role in chlorophyll degradation, growth, and development.

## 2. Results

### 2.1. The Isolation and Analysis of the Gene ZjNOL 

The DNA fragment comprising the *ZjNOL* coding domain sequence (CDS) was cloned and ligated into cloning vector pMD19-T, according to the Zoysia Genome Database [13]. The *ZjNOL* gene has a 981-bp ORF that codes for a 326-amino-acid protein, which belongs to the SDR superfamily. The molecular weight of ZjNOL is 26.62 kD, and the theoretical isoelectric point is 10.76. *ZjNOL* had 12 exons (Supplementary Figure S1). 

**Figure 2:**
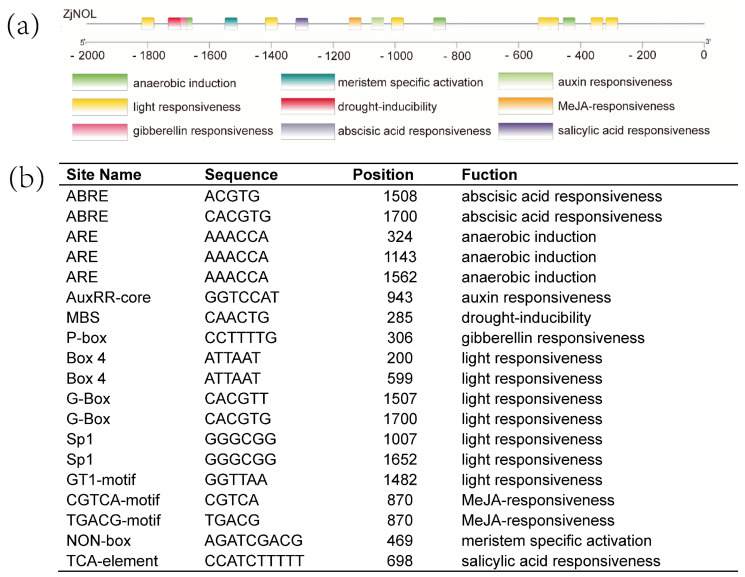
Analysis of upstream sequence of *ZjNOL* gene. (**a**) Cis-acting elements upstream of *ZjNOL* gene. (**b**) List of predicted binding sites for the transcription factors upstream of *ZjNOL* gene.

The upstream sequence was used to investigate the *cis*-regulatory elements of *ZjNOL*. Multiple photoresponsive elements, such as BOX4, G-box, Sp1, and GT1 motif, are found in the upstream region of *ZjNOL* (Figure 2). Several hormone-related response elements (ABRE, AUXRR-core, P-box CGTCA-motif, TGACG-motif, TCA-element) are found, which are involved in the pathways of abscisic acid (ABA), 3-indoleacetic acid (IAA), gibberellin (GA), methyl jasmonate (MeJA), and salicylic acid (SA). In addition, various *cis*-acting elements have been found that are involved in growth and development as well as stress tolerance, such as ARE, MBS, and non-Box.

### 2.2. ZjNOL Expression Profiles and Expression Analysis of AsZDS, AsNCED, AsCLH1 and AsNYC Induced by Dark

**Figure 3:**
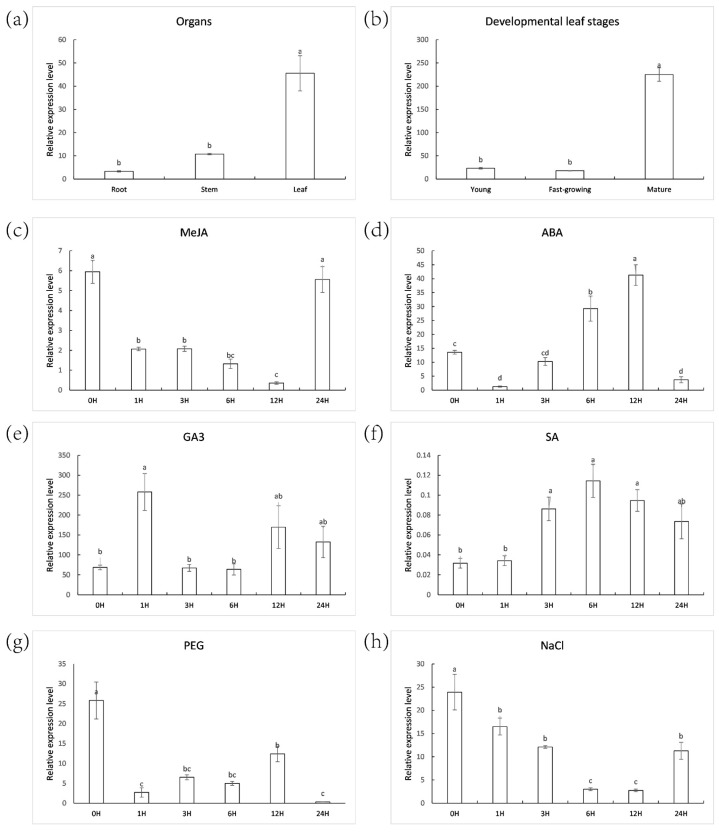
*ZjNOL* expression profiles. (**a**) Expression pattern of *ZjNOL* in the root, stem, and leaf. (**b**) ZjNOL expression pattern in leaves at various phases of development. (**c**–**f**) Leaf expression pattern after 10 mM MeJA treatment (**c**), 10 mM ABA treatment (**d**), 10 μM GA3 treatment (**e**), 0.5 mM SA treatment (**f**). (**g**,**h**) Leaf expression analysis of ZjNOL after 20% PEG4000 (**g**), 300 mM NaCl (**h**). Significant differences (*p* < 0.05, *n* = 3) are shown by different letters above the columns.

Real-time PCR was used to detect *ZjNOL* expression patterns in various tissues and developmental phases. The results revealed that *ZjNOL* was expressed in root, stem, and leaf tissues, with leaf expression being substantially higher (Figure 3a). Among all the detected tissues, *ZjNOL* was expressed highest in mature leaves, which was 9.67 and 12.61 times of those in young and fast-growing leaves, respectively (Figure 3b). *ZjNOL* expression was up-regulated by ABA or SA (Figure 3c–f). The level of *ZjNOL* expression was reduced after MeJA treatment (Figure 3c–f). The expression level of *ZjNOL* rose and then dropped after gibberellic acid (GA3) therapy. The expression of *ZjNOL* dropped initially and then rose in response to salt and drought treatments. *ZjNOL* may be involved in leaf growth, stress, and hormone response.

**Figure 4:**
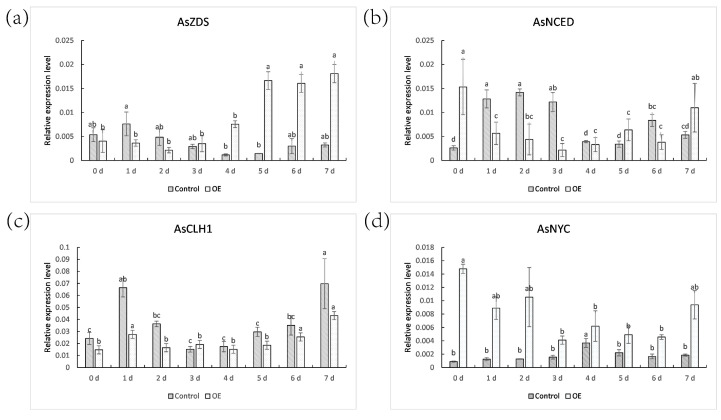
Expression levels of *AsZDS* (**a**), *AsNCED* (**b**), *AsCLH1* (**c**), *AsNYC* (**d**) in control and OE subjected to dark at 0–7 days. The control (grey) and OE (white) columns were separately compared using one-way ANOVA with Duncan test. Significant differences (*p* < 0.05, *n* = 3) are shown by different letters above the columns.

To understand the role of *ZjNOL* in plant growth and development, the expression profile of *AsZDS* (Z-carotene desaturase), *AsNCED* (9-cis-epoxyoid dioxygenase), *AsCLH1* (chlorophyllase 1), and *AsNYC* (non-yellow coloring) after dark treatment was studied (Figure 4). *AsNYC* and *AsCLH1* were confirmed to play important roles in the degradation of chlorophyll [10,14]. *AsZDS* and *AsNCED* were reported to be involved in carotenoid metabolism [15,16]. After dark treatment, *AsZDS* did not change in the control plants, but in OE plants, the expression level of *AsZDS* increased from the fifth day, reaching three times of that in the first day. *AsNCED* in OE plants had a tendency of first reducing and then increasing, whereas it exhibited a trend of first increasing and then decreasing in the control plants. The expression pattern of AsCLH1 was similar in the control and OE plants. Under dark treatment, AsNYC expression in the control remained stable; however, AsNYC expression in OE had a declining trend. 

### 2.3. Analysis of the Protein ZjNOL

**Figure 5:**
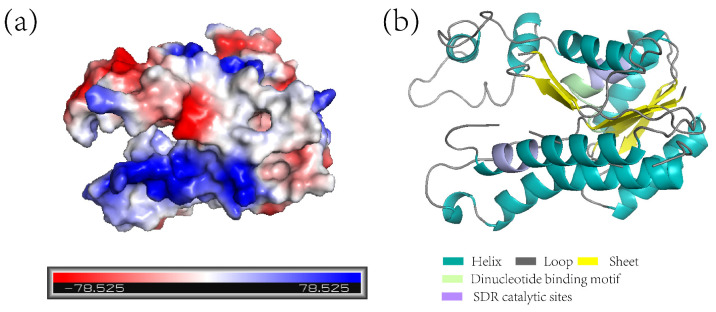
ZjNOL protein analysis. (**a**) The electrostatic potential distribution of ZjNOL protein. The value of electrostatic potential is positively associated with the depth of color, with blue representing positive potential and red representing negative potential. (**b**) Protein structure simulation of ZjNOL.

A modelled 3D tertiary structure analysis demonstrated that there was a negative potential area on the surface of the ZjNOL protein, primarily near the helix structure (Figure 5a,b). ZjNOL has a dinucleotide binding motif (TGXXXGXG) and SDR catalytic site (YXXXK) at the position of amino acids 88 and 211 (Figure 5b).

NOL proteins were phylogenetically analyzed using the neighbor-joining method and protein sequences from various species obtained from the NCBI database (Supplementary Figure S2A,B). The phylogenetic tree reflects the valid taxonomy of the included species. ZjNOL has the most homology with NOL of Zea mays and all the other monocots above them. Although AtNOL does not have the highest resemblance with AtNOL from Arabidopsis thaliana, their motif architectures are similar (Supplementary Figure S2B).

### 2.4. Subcellular Localization of ZjNOL 

**Figure 6:**
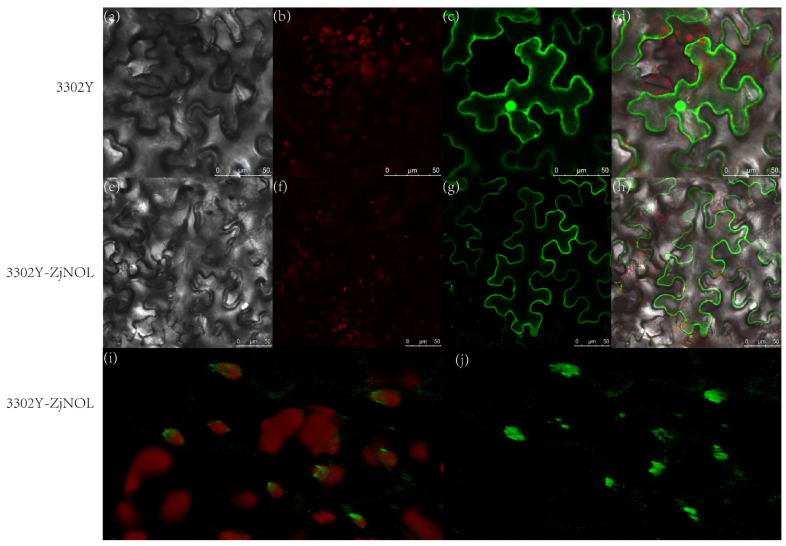
Subcellular localization of ZjNOL in cells. (**a**–**d**) YFP fluorescent detection. (**e**–**j**) Fusion protein of ZjNOL and YFP fluorescent detection. (**a**,**e**): Bright light; (**b**,**f**): chlorophyll autofluorescence; (**c**,**g**,**j**): YFP fluorescence; (**d**,**h**,**i**): merged signal.

After agrobacterium-mediated tobacco leaf infection, yellow fluorescent protein (YFP) fluorescence signal overlapped with chlorophyll self-fluorescence in the chloroplasts in the leaf tissue with transient expression of YFP and ZjNOL fusion protein, and the fluorescence signal was also found in the cytoplasm (Figure 6a–j). As the control, untargeted YFP was localized in the whole cell. According to subcellular localization assays, ZjNOL was mostly localized in the chloroplast and cytoplasm. ZjNOL was not equally distributed inside the chloroplast (Figure 6i,j).

### 2.5. ZjNOL Interacts with ZjNYC

**Figure 7:**
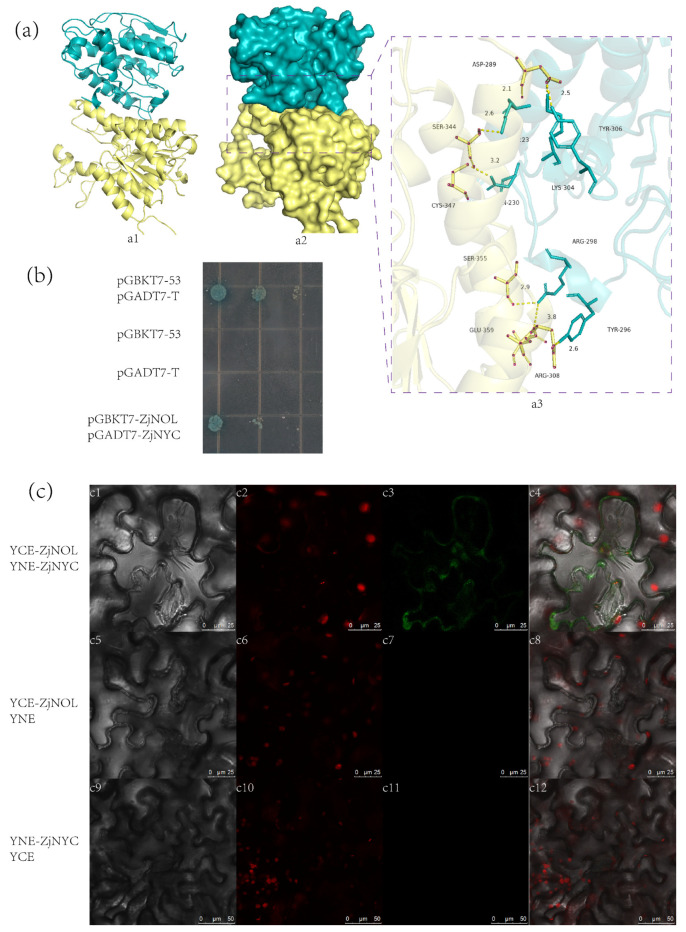
The interaction between ZjNOL and ZjNYC. (**a**) The binding mode of the complex ZjNYC with ZjNOL. (**a1**), The backbone of protein was rendered in the tube and colored in blue (ZjNYC) and yellow (ZjNOL). (**a2**), ZjNOL and ZjNYC proteins were rendered by the surface. (**a3**), The detailed binding mode of ZjNOL with ZjNYC. Yellow dash represents hydrogen bond or salt bridge. (**b**) Interaction between ZjNOL and ZjNYC. By 10-fold repeated dilutions, yeast cells were identified on increasing stringency QDO/X/A agar plates. Positive clones were blue, whereas negative clones were white or missing. The negative control was pGBKT7-53 or pGADT7-T, while the positive control was pGBKT7-53 and pGADT7-T co-transformed. (**c**) The interaction between ZjNOL and ZjNYC in living cells showed by BiFC. (**c1**–**c4**), YCE-ZjNOL and YNE-ZjNYC fluorescent detection. (**c5**–**c8**), YCE-ZjNOL and YNE fluorescent detection. (**c9**–**c12**), YNE-ZjNYC and YCE fluorescent detection. (**c1**,**c5**,**c9**), Bright light; (**c2**,**c6**,**c10**), chlorophyll autofluorescence; (**c3**,**c7**,**c11**), YFP fluorescence; (**c4**,**c8**,**c12**), merged signal. Negative control was YCE-ZjNOL co-expression with YNE or YNE-ZjNYC co-expression with YCE.

For visualization, the conformation of ZjNOL and ZjNYC proteins with the highest negative energy was chosen. ZjNOL had a binding score of −310.52 kcal/mol to ZjNYC protein, and its binding sites comprised Arg-298, Tyr-306, Lys-304, Lys-223, Gln-230, Tyr-296, and other amino acid residues. Glu-359, Ser-355, Asp-289, Ser-344, Cys-347, Arg-308 and other amino acid residues are among the binding sites of ZjNYC protein. The contact residues of ZjNYC and ZjNOL proteins can create a number of interactions, including salt bridges, hydrogen bonds, hydrophobic interactions, and others (Figure 7a). These interactions can help to increase the stability of the ZjNYC and ZjNOL protein complex. Furthermore, the binding surface diagrams of the two proteins revealed that the ZjNOL protein surface matched well with the ZjNYC protein surface, facilitating the creation of stable binding (Figure 7a).

The connection between ZjNOL and ZjNYC proteins was investigated using yeast two-hybrid and bimolecular fluorescence complementation (BiFC) methods. The results showed that the yeast cells pGADT7-ZjNYC and PGBKT7-ZjNOL developed normally and appeared blue on the QDO/X/A plate, indicating that they can interact in the yeast cell (Figure 7b). ZjNOL and ZjNYC were fused to the C-terminal and N-terminal of YFP, respectively, and were expressed in tobacco leaf cells by co-transformation. After 3-day culture in the dark, a strong YFP signal was found in the chloroplasts and cytoplasm, showing how the two proteins physically interacted in living plant cells. These findings confirmed that ZjNOL interacts with the ZjNYC protein.

### 2.6. Ectopic Expression of ZjNOL Resulted in Leaf Yellowing and Sluggish Development

**Figure 8:**
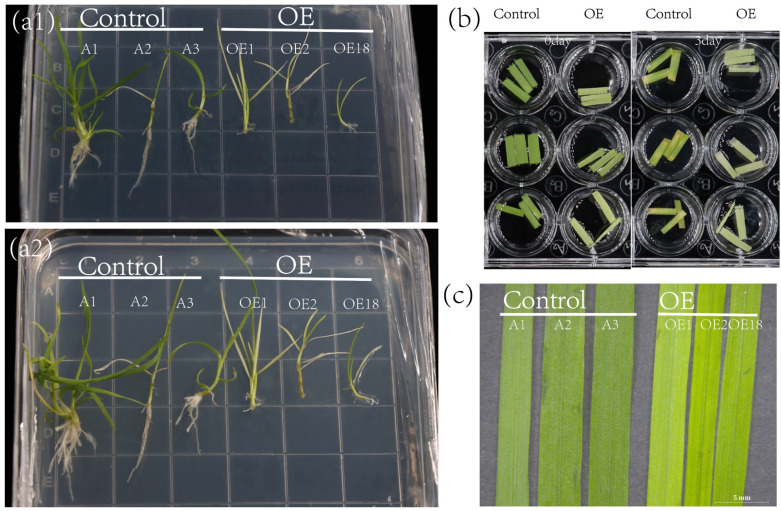
Leaf yellowing phenotype of transgenic creeping bentgrass. (**a1**,**a2**) Comparison of transgenic creeping bentgrass (OE) and control on a MS plate. (**b**) Detached leaves of OE and control at 30-days-old were soaked in 3 mM MES for 0 or 3 days. (**c**) Comparison of the phenotypes of transgenic creeping bentgrass and control at 30 days.

Transgenic creeping bentgrass with *ZjNOL* was obtained by the *Agrobacterium tumefaciens* mediated method. The control group was named control, while the *ZjNOL* ectopic overexpression creeping bentgrass was named OE. The transgenic plants had yellow leaves, indicating that ectopic expression of *ZjNOL* may result in a reduction in chlorophyll concentration. Additionally, the root length of OE was less than that of the control. Those findings imply that *ZjNOL* has negative effect on the development of cholophyll biosynthesis and leaf color (Figure 8).

### 2.7. Morphological Changes of Transgenic Plants

**Figure 9:**
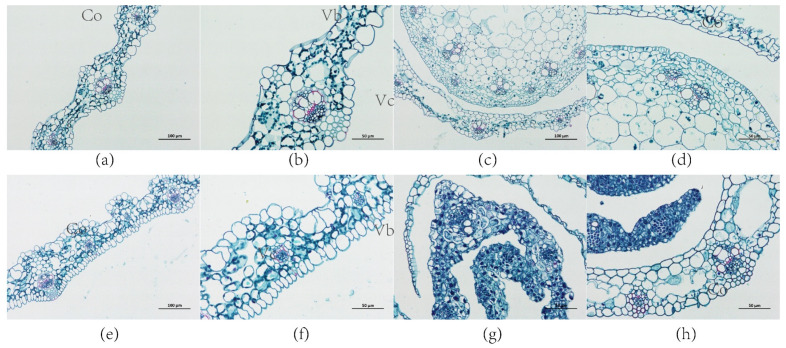
Morphological observation of leaves and stems with saffron solid green stain. (**a**,**b**) Leaf of control group; (**e**,**f**) leaf of transgenic plant; (**c**,**d**) stem of control group; (**g**,**h**) stem of transgenic plant; (**a**,**c**,**e**), 200×, scale bar = 100 μm; (**b**,**d**,**f**,**g**,**h**), 400×, scale bar = 50 μm. Co, cortex; Vc, vascular column; Vb, vascular bundle. Pi, pith.

Ferro green was used to stain the leaves of the control and transgenic plants, while ferro red was used to stain the cell walls, which were rich in cellulose. In the leaves of the transgenic plants, cortex tissue thickness and cell length were increased. The cortex of transgenic plants was thicker and the vascular column cells were smaller than those in the control (Figure 9). In the cross sections of the control and transgenic stems, the average length of vascular column cells was 54.52 μm and 31.71 μm, respectively. Additionally, the average pith cell length of the control and transgenic plants was 27.14 μm and 17.34 μm, respectively (Supplementary Figure S3). The leaves and stems of the transgenic plants were more likely to be colored green by ferro solid green staining in the cross sections, indicating that they had developed more cellulose in their cell walls.

### 2.8. Expression of ZjNOL Gene Results in Chloroplast Morphological Changes

**Figure 10:**
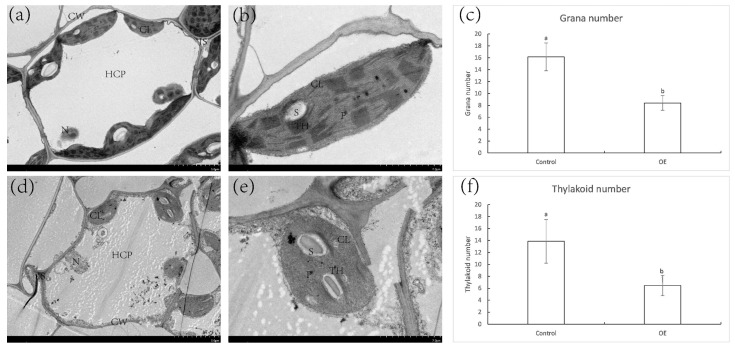
Changes in cell and chloroplast structure in leaves of control and OE. (**a**,**b**) Transmission electron microscopy analysis of cells and chloroplast in control leaves; (**d**,**e**) transmission electron microscopy analysis of cells and chloroplast in OE leaves. (**c**,**f**) Grana number and thylakoid number of chloroplasts in control and OE cells. Different letters above the columns indicate significant differences (*p* < 0.05, *n* = 20). (**a**,**d**), Scale bar = 5 μm; (**b**,**e**), scale bar = 2 μm. CL, chloroplast; CW, cell wall; HCP, huge celled parenchyma; IS, intercellular space; N, nucleus; P, plastglobulus; S, starch granule; TH, thylakoid.

The chloroplasts of OE shifted from a typical spindle shape to an ellipsoidal shape. In comparison to the chloroplasts of the control, the length and width of chloroplasts in OE grew shorter and wider. Furthermore, the average number of grana stacks (GS) in OE cells was significantly lower than those in the control cells (Figure 10). The grana in the chloroplasts of the control leaf cells were well developed and highly stacked, with an average of 13.85 thylakoids per grana layer (Figure 10c). In the chloroplasts of OE, the thylakoid network was plainly incomplete. The number of GS in chloroplasts was reduced to 4–14, and each GS layer contained an average of 6.45 thylakoids (Figure 10f). Some thylakoids developed into long bundles of pseudograna, resulting in essentially the unstacking of the grana and an incompact thylakoid structure that was loose in the chloroplast of OE leaves. 

### 2.9. Net Photosynthetic Rate, Fv/Fm, Chlorophyll Content, Membrane Ion Leakage of Control and OE

**Figure 11:**
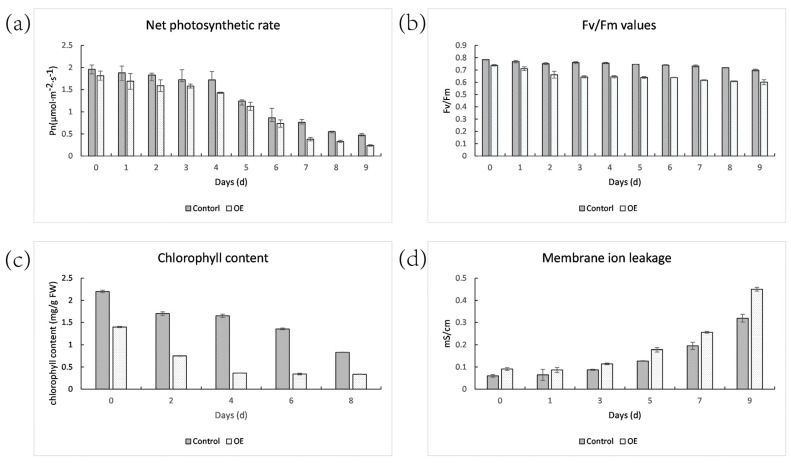
Physiological changes in control and OE leaves during senescence induced by dark. (**a**) Net photosynthetic rate changes during dark treatment. (**b**) Fv/Fm value changes. (**c**) Chlorophyll content changes. (**d**) Membrane ion leakage changes.

The leaves of OE plants had lower net photosynthetic efficiency and Fv/Fm values than the control plants. The net photosynthetic rate of the control and OE plants declined by 75.91% and 86.83%, respectively, after 9 days of dark treatment (Figure 11). The Fv/Fm values of the control and OE plant reduced by 10.86% and 18.72%, respectively. After 8 days of dark treatment, the chlorophyll content of the OE reduced by 75.90%, whereas the control only decreased by 62.13%. Furthermore, OE plants demonstrated a higher rate of ion leakage. This meant that following shading, the photosynthetic level of OE plants reduced faster.

### 2.10. Hormone Content

**Figure 12:**
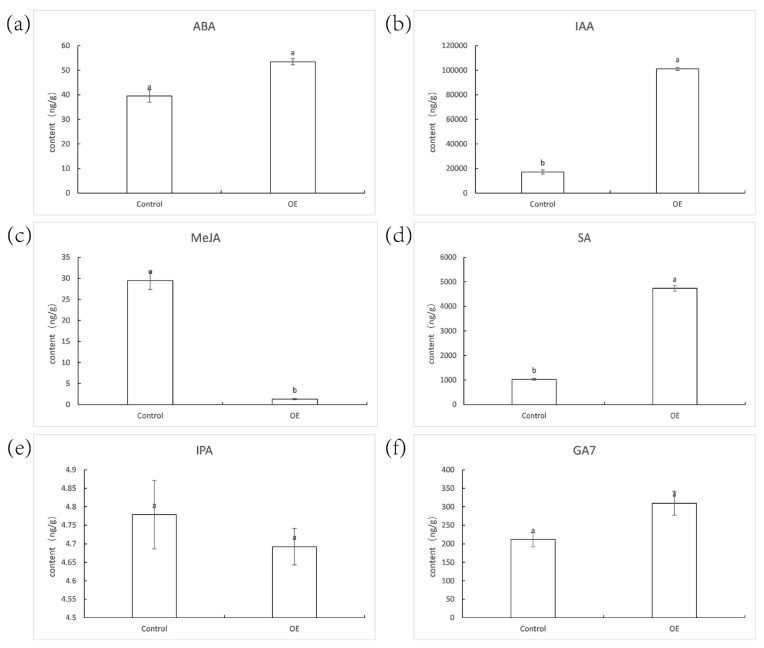
The contents of the plant hormones ABA (**a**), IAA (**b**), MeJA (**c**), SA (**d**), IPA (**e**), and GA_7_ (**f**) were determined. Significant differences (*p* < 0.05, *n* = 3) are shown by different letters above the columns.

There were no significant variations in the hormone content of ABA, IPA, and GA7 between the control and OE plants (Figure 12). The IAA and SA concentrations in OE were 5.89 and 4.60 times more than in the control, respectively. Simultaneously, the MeJA contents decreased drastically in the transgenic plants, only 4% of the control.

### 2.11. The Transcriptome Analysis

**Figure 13:**
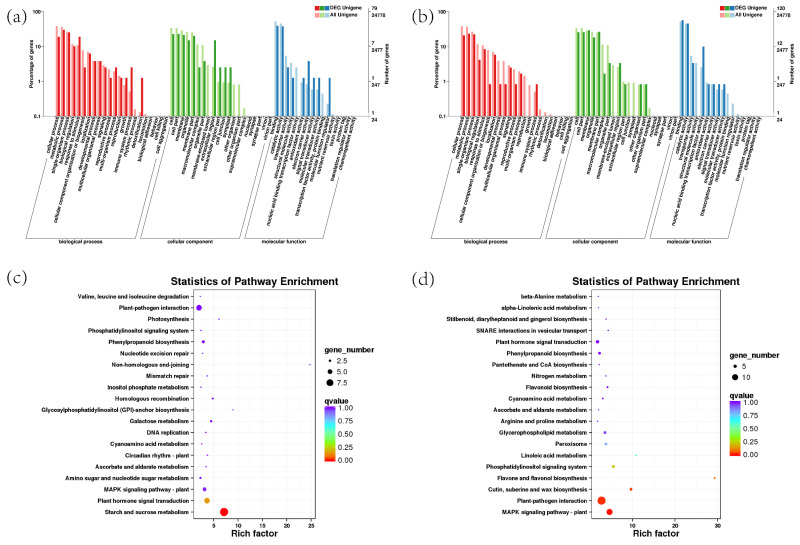
GO and KEGG enrichment analyses for the DEGs. (**a**,**b**) GO analysis of up-regulated and down-regulated DEGs; (**c**,**d**) KEGG analysis of up-regulated and down-regulated DEGs; significant enrichment was assumed for GO enrichment terms and KEGG enrichment terms, with a *p*-value of 0.05.

Transcriptome sequencing was applied and a total of 296 differentially expressed genes (DEGs) was identified between the transgenic and control samples, of which 133 were up-regulated and 163 were down-regulated. For example, senily-associated protein gene *DIN1* (C153676.graph_c0) and *leucine tRNA ligase* gene (C163435. Graph_c1) were significantly up-regulated. *NADH-ubiquinone oxidoreductase chain 4* gene (c152156.graph_c1), *pheophytinase* gene (c159122.graph_c0) in the chloroplast part (GO:0044434), chloroplast stroma (GO:0009570), chloroplast thylakoid membrane (GO:0009535) and chloroplast thylakoid (GO:0009534) were significantly down-regulated. The up-regulated DEGs were assigned into 28 pathways and the most enriched pathways were “starch and sucrose metabolism” and “plant hormone signal transduction”. Eight pathways were enriched for down-regulated DEGs, with “MAPK signaling pathway” being the most enriched (Figure 13). A total of ten genes were selected for quantitative RT-PCR assays. The expression patterns of the genes acquired from the RNA-seq data were compared to those obtained from qRT-PCR, and the findings show that the expression trends of these 10 genes in RT-qPCR were compatible with those found by RNA-Seq analysis (Supplementary Table S1).

## 3. Discussion

The *ZjNOL* gene encodes a SDR superfamily protein. The SDR family contains a significant number of proteins that are split into 464 groups [17]. SDR proteins have a wide range of functions and low consistency, typically between 20% and 30% [18]. The SDR family of proteins are all based on the Rossmann-fold structure, with an N-terminal dinucleotide cofactor binding motif and an active site with a catalytical residue motif [19]. The three-dimensional structure prediction revealed that ZjNOL protein contains Rossmann-fold, a structure that the central β folded and surrounded by three α helixes. At amino acid 80, ZjNOL has the dinucleotide binding motif (TGXXXGXG) and SDR catalytic site (YXXXK) at amino acids 88 and 211. Dinucleotide binding motifs can act as a coenzyme binding region’s binding motif [20]. It is less likely that the ZjNOL protein’s N-terminal region contains a transmembrane domain (Supplementary Figure S4). To determine ZjNOL’s intracellular localization, we fused YFP to ZjNOL and performed subcellular localization of tobacco. The results indicated that ZjNOL was found in the stromal side of the thylakoid membrane. In addition, the heterologous expression of NOL may impact plant growth and development, which may be a result of the lower photosynthetic efficiency resulting from chlorophyll degradation, which requires further investigation [9,11].

**Figure 14:**
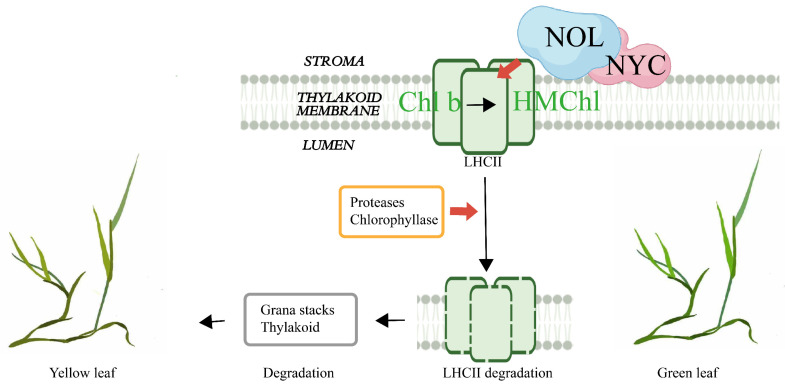
NOL regulates the degradation of chlorophyll and LHCII during leaf senescence [10].

Proteins in the SDR family commonly form dimers or tetramers [19]. Studies in rice confirmed that NYC1 and NOL may form a complex and function as chlorophyll *b* reductase (Figure 14) [10]. Only until Chl b is converted to HMChl a by the NYC–NOL complex can LHCII be degraded by protease, and then chlorophyllase can hydrolyze chlorophyll produced from LHCII. In addition, the degradation of LHCII promoted the degradation of the grana structure and thylakoid (Figure 10 and Figure 14). The findings of the ZjNOL and ZjNYC interaction research revealed that the two proteins were able to develop stable binding and maintain good matching. In this study, the BiFC and transmission electron microscopy results revealed that ZjNYC and ZjNOL co-locate and the ectopic expression of *ZjNOL* may also induce the deterioration of the grana structure and thylakoid. In transgenic plants, the vascular bundle cells shrank considerably, which may be connected to morphological changes caused by chlorophyll degradation. 

Although the promoter element analysis revealed that the *ZjNOL* promoter contains cis-elements linked with ABA, IAA, GA, MeJA, and SA, the hormone content analysis revealed no significant variation in ABA and GA_7_ content between the transgenic and control plants. The IAA and SA concentrations were substantially greater in the transgenic plants than in the control, while MeJA concentrations were significantly lower in transgenic plants. To investigate the effect of *ZjNOL* ectopic expression on leaf color, we examined the effect of darkness treatment on the expression of the *AsZDS*, *AsNCED*, *AsCLH*, and *AsNYC* genes. *AtZDS* regulates the catalytic impact of Z-carotene on tetracis-lycopene and is also a key gene for carotenoids synthesis [15]. *AsNCED*, a carotene lyase, is a critical enzyme for stress-induced ABA biosynthesis [16]. *AsCLH* catalyzes the ester bond hydrolysis of chlorophyll and chlorophyl, the initial step in the breakdown of chlorophyll [14]. *AsNYC* participates in the breakdown of the LHCII [6]. The relative expression of AsCLH1 between the control and OE plants have similar trends on different days. Conversely, the trends of overexpression of AsZDS, AsNCED, and AsNYC are predominantly distinct with the control plants. The impact of ectopic expression of *ZjNOL* on plant leaf color may be due to a variety of biological processes, not just chlorophyll. Fewer differentially expressed genes were filtered out in the transcriptome, which may be attributable to the fact that the plant samples for transcriptome analysis had not yet passed the senescence stage. This shows that *NOL* performs a major regulatory function exclusively in mature leaves, which is congruent with the findings of expression pattern analysis. Even yet, numerous genes associated with chlorophyll degradation were screened by transcriptome sequencing, and *DIN1* and *LARS* (*leucine tRNA ligase*) were considerably up-regulated in transgenic plants. *Mt-nd4* (*NADH-ubiquinone oxidoreductase chain 4*) and *PPH* (*pheophytinase*) were dramatically down-regulated. *DIN1* is a dark-induced senescence-related gene encoding the synthesis of senily-associated protein DIN1. *DIN1* encoded peptides may be imported by isolated chloroplasts and subsequently localized to the stromal fraction in *Raphanus sativus*, and it was thought to be the first gene producing a chloroplast protein that is negatively regulated by light [21]. *LARS* is targeted to chloroplasts. Chloroplast protein hydrolysis increased the amounts of amino acids during the dark treatment in *Camellia sinensis* [22]. The research suggested that aminoacyl tRNA synthetases of *Oryza sativa* may govern amino acid metabolism and the homeostasis of chloroplast thylakoid growth via regulating the protein synthesis process [23]. The NADH–ubiquinone oxidoreductase complex is the principal entry point of electrons into the respiratory chain and it is regarded to be directly associated with sustaining effective flow underambient (photorespiratory) circumstances in *Arabidopsis thaliana* [24]. PPH of *Solanum lycopersicum* in particular dephytylates pheophytin, leading to the creation of pheophorbide a, a crucial step in the chlorophyll breakdown process [25]. PPH catalyzes the hydrolysis of chlorophyll to chlorophyllide and phytol in *Arabidopsis thaliana*, which was considered to be the rate-limiting step of the pathway [26]. Heterologously expressed PPH dephytylates phein to pheide but does not accept chlorophyll as a substrate. PPH can only dehydrogenate phenol without taking chlorophyll as a substrate and has no activity with chlorophyll [26]. This indicates that PPH must conduct its chlorophyll degrading function after NOL has fulfilled its role. However, the underlying chemical mechanism driving the high substrate selectivity of PPH towards pheophytin remains unexplained in *Arabidopsis thaliana* [27].

## 4. Materials and Methods

### 4.1. Plant Materials and Growth Conditions

The plants of *Zoysia japonica* (cv. Compadre), *Agrostis stolonifera* (cv. Penn A-4), *Arabidopsis thaliana* and *Nicotiana benthamiana* were cultivated in pots, which were incubated in a plant growth chamber (26/20 °C, 50% humidity, 16 h/8 h photoperiod). All the cultivated species are from and kept in a laboratory. Transgenic plants of 4-weeks-old were transferred to dark environments for 8 d consecutively to perform dark treatments [28]. 

### 4.2. Gene Cloning and Vector Construction

Healthy *Zoysia japonica* was selected as the experimental material. Total RNA was isolated from 3-month-old leaves according to the manufacturer’s recommendations (Promega, Madison, WI, USA, plant RNA isolation kit). The full-length cDNAs of ZjNOL were amplified according to the Zoysia genome database [13], and then ligated into the vectors pDM19-T, 3302Y, 3302YUBI, pGBKT7 and YCE4 with the primers listed in Supplemental Table S2. The plasmid 3302Y-ZjNOL was subsequently transformed into the *Agrobacterium tumefaciens* strain ‘GV3101’. The coding sequences of *ZjNYC* were cloned into pGADT7 and YNE vectors according to the Zoysia genome database.

### 4.3. Generation of Transgenic Plants

The plant expression vector 3302YUBI-ZjNOL was transformed into Agrobacterium EHA105 [29]. The Agrobacterium-mediated method was conducted following the protocol described by Luo et al. [30]. The transgenic seedlings of *Agrostis stolonifera* were identified on selective agar plates and transferred to the controlled growth chamber conditions and subsequent experiments were conducted. Transgenic plants carrying 3302YUBI were used as a negative control. 

### 4.4. Subcellular Localization and BiFC Analysis

The YFP plasmid, ZjNOL and YFP recombinant plasmid 3302Y-ZjNOL were respectively introduced into agrobacterium strain EHA105, then infiltrated young leaves of tobacco as previously described [31]. The interaction between ZjNOL and ZjNYC was verified using the BiFC approach [32]. The plasmids YCE4-ZjNOL and NE6-ZjNYC, which contain THE C-terminal and N-terminal of YFP protein, were transformed with Agrobacterium and co-infected tobacco. A Leica TCS SP 8 confocal microscope was used to capture the YFP fluorescence signal.

### 4.5. Yeast Two-Hybrid Assay

The PGBKT7-ZjNOL and PGADT7-ZjNYC recombinant plasmids were transformed into yeast strains Y2H and Y187, respectively, using the Yeastmaker™ Yeast Transformation System (Clontech Laboratories, Mountain View, CA, USA). The MATCHMAKER GAL4 yeast two-hybrid system was set up according to the manufacturer’s instructions for the Matchmaker^TM^ Gold Yeast Two-Hybrid System (Clontech Laboratories). 

### 4.6. Measurement of Net Photosynthesis of Leaves

The net photosynthetic rate was measured using the Li-6400 portable photosynthetic system (Li-Cor Biosciences, Lincoln, NE, USA). The artificial light utilized for the measurement was 1200 μmol m^−2^ s^−1^, and three repeats were carried out to verify the uniformity of the test data. All measurements were taken between 10 and 12 a.m.

### 4.7. Photosynthetic Pigment Analysis and Electrolyte Leakage (EL) Measurement

Pigment and ion permeability were measured in the leaves treated with darkness for 0–7 days. Total chlorophyll was extracted from the 2nd leaves of the control and transgenic plants, quantified spectrophotometrically as described previously [33]. Leaf membrane stability was assessed by measuring EL following the protocol described earlier [34]. 

### 4.8. Transmission Electron Microscopy

Transmission electron microscopy experiments were performed on the leaves of the control and transgenic plants. The second leaf of the transgenic and wild-type plants was chosen and immersed in a fixed buffer solution for 24 h. The specimens were subsequently stained and observed as described previously [35]. 

### 4.9. Morphological Observation 

The second leaf and stem of the transgenic plants of 30-days-old were selected for ferro red solid-green staining. The walls of lignified cells were stained red, while the walls of fibrotic cells were stained green. Under a light microscope, the samples were observed and the images were captured [36]. 

### 4.10. Bioinformatics Analysis

Fifteen NOL proteins (Supplementary Table S3) from different plant species were obtained through homolog identification by BLAST from the NCBI database. Multiple sequence alignments were used with GeneDoc and ClustalX version 2.1 with default settings. The amino acid sequences of NOLs were used for motif analysis through MEME (http://meme-suite.org/tools/meme (accessed on 20 October 2021)) and TBtools, and phylogenetic tree analysis through TBtools and MEGA version 6.0, with the neighbor-joining (NJ) method. ZjNOL gDNA structures were constructed using GSDS 2.0 (http://gsds.gao-lab.org/Gsds_about.php (accessed on 18 November 2021)) [37]. The *cis*-regulatory elements analysis used the PlantCARE database (http://bioinformatics.psb.ugent.be/webtools/plantcare/html/ (accessed on 18 November 2021)) and TBtools [38,39,40,41]. The secondary structure and tertiary structure of the proteins were analyzed using the Compute pI/MW tool (http://web.expasy.org/compute_pi/ (accessed on 5 May 2021)), Phyre v2.0 tool (www.sbg.bio.ic.ac.uk/phyre2/ (accessed on 05 May 2021)), HDOCK (http://hdock.phys.hust.edu.cn/ (accessed on 1 March 2021)) and PyMOL version 2.5.2 [42].

### 4.11. Expression Levels of ZjNOL Gene and Pigment-Related Genes

RT-PCR (real-time quantitative PCR) was used to evaluate the expression pattern of the *ZjNOL*. Different tissues (roots, stems, and leaves) of 2-month-old *Zoysia japonica*, as well as leaves from *Zoysia japonica* at various growth stages (young, fast growing, mature), were collected. A total of 10 μmol/L ABA, 10 μmol/L MeJA, 200 μmol/L ETH, 300 mmol/L NaCl, and 20% PEG4000 were used to treat *Zoysia japonica*. RNA was extracted and cDNA was produced from the leaves at 0 h, 1 h, 3 h, 6 h, 12 h, and 24 h after treatment. The Z. japonica beta-actin was used as an internal reference gene (GenBank accession number: GU290546) [43]. RNA was isolated from OE and control plants that had been exposed to darkness for 0–7 days. To evaluate the relative expression levels of *AsZDS*, *AsNCED*, *AsCLH1*, and *AsNYC*, the actin gene (GenBank accession number: DY543529) was utilized as an internal control [44]. Three biological replicates were set for each treatment. The sequences of related primers used above are shown in Supplementary Table S2. The relative expression levels were calculated using the 2^−ΔΔCt^ method [45].

### 4.12. Hormone Contents Assay

One-month-old leaves of the control and OE plants were collected and ground after quick-freezing with liquid nitrogen. The metabolites were extracted and concentrated and redissolved with 80% methanol/aqueous solution for LC-MS/MS analysis. The experiment was carried out using an AB SCIEX 6500+ Qtrap mass spectrometer [46,47].

### 4.13. Transcriptomic Analysis

The leaves of 20-days-old from OE (OE1, OE3, OE18) and control plants (A1, A2, A3) were analyzed by RNA-Seq. Library construction, sequencing, differential expression gene screening and transcriptome analysis were performed as described previously [48]. GO and KEGG analysis were performed using BMKCloud (www.biocloud.net).

### 4.14. Experimental Validation of DEGs by RT-PCR

The transcriptome analysis-derived DEGs were validated using RT-PCR. Ten genes (*AsFEH*, *AsTPS*, *AsCK1*, *AsYLS3*, *AsJRL9*, *AsWRKY23*, *AsLOX*, *AsERF9*, *AsFAR1*, *AsERF4*) were chosen at random for RT-PCR, and the experimental procedure was as described before. The results of the gene expression levels were then analyzed by the corresponding standard deviations repeated by the three techniques.

## 5. Conclusions

The NYC-like enzyme (NOL) is a key enzyme in the degradation of chlorophyll *b*. The *NOL* gene of *Zoysia japonica* codes a protein with 326 amino acids and has a molecular mass of 26.62 kD. ZjNOL is found on the stromal side of the thylakoid membrane, and both ZjNOL and ZjNYC proteins are capable of forming stable structures and interacting among each other. Ectopically expressed *ZjNOL* resulted in yellow leaves, a larger cortex, and smaller vascular column cells in creeping bentgrass. Additionally, the transgenic plants revealed morphological changes in their chloroplast structure, with a significant reduction in the number of grana and thylakoids per grana stack. The features and activities of *ZjNOL* will serve as a starting point for further study into the gene’s functions and regulatory mechanisms.

## Data Availability

The data presented in this study are available on request from the corresponding author.

## Supplementary Materials

The following supporting information can be downloaded at: https://www.mdpi.com/article/10.3390/ijms23116032/s1.

## References

[1] Hörtensteiner S. (2004). The Loss of Green Color during Chlorophyll Degradation—A Prerequisite to Prevent Cell Death?. Planta.

[2] KiJhlbrandt W. (1994). Structure and Function of the Plant Light-Harvesting Complex, LHC-II. Curr. Opin. Struct. Biol..

[3] Allen J.F., Forsberg J. (2001). Molecular Recognition in Thylakoid Structure and Function. Trends Plant Sci..

[4] Sakuraba Y., Kim Y.S., Yoo S.C., H?Rtensteiner S., Paek N.C. (2013). 7-Hydroxymethyl Chlorophyll a Reductase Functions in Metabolic Channeling of Chlorophyll Breakdown Intermediates during Leaf Senescence. Biochem. Biophys. Res. Commun..

[5] Sakuraba Y., Schelbert S., Park S.Y., Han S.H., Lee B.D., Andres C.B., Kessler F., Hortensteiner S., Paek N.C. (2012). STAY-GREEN and Chlorophyll Catabolic Enzymes Interact at Light-Harvesting Complex II for Chlorophyll Detoxification during Leaf Senescence in Arabidopsis. Plant Cell.

[6] Rubina J., Sullivan K.L., Ross C., Erridge Z.A., David C., Mclachlan A.R.G., Brummell D.A., Dijkwel P.P., Hunter D.A. (2015). Staying Green Postharvest: How Three Mutations in the Arabidopsis Chlorophyll *b* Reductase Gene *NYC1* Delay Degreening by Distinct Mechanisms. J. Exp. Bot..

[7] Luo F., Cheng S.C., Cai J.H., Wei B.D., Zhou X., Zhou Q., Zhao Y.B., Ji S.J. (2019). Chlorophyll Degradation and Carotenoid Biosynthetic Pathways: Gene Expression and Pigment Content in Broccoli during Yellowing. Food Chem..

[8] HRtensteiner S., Vicentini F., Matile P. (1995). Chlorophyll Breakdown in Senescent Leaves: Enzymatic Cleavage of Pheophorbide a In Vitro. New Phytol..

[9] Jia T., Ito H., Tanaka A. (2015). The Chlorophyll *b* Reductase NOL Participates in Regulating the Antenna Size of Photosystem II in *Arabidopsis thaliana*. Procedia Chem..

[10] Sato Y., Morita R., Katsuma S., Nishimura M., Kusaba M. (2010). Two Short-Chain Dehydrogenase/Reductases, NON-YELLOW COLORING 1 and NYC1-LIKE, Are Required for Chlorophyll *b* and Light-Harvesting Complex II Degradation during Senescence in Rice. Plant J..

[11] Yu G., Xie Z., Zhang J., Lei S., Lin W., Xu B., Huang B. (2021). *NOL*-mediated Functional Stay-green Traits in Perennial Ryegrass (*Lolium Perenne* L.) Involving Multifaceted Molecular Factors and Metabolic Pathways Regulating Leaf Senescence. Plant J..

[12] Xu Y., Huang B. (2018). Comparative Transcriptomic Analysis Reveals Common Molecular Factors Responsive to Heat and Drought Stress in *Agrostis stolonifera*. Sci. Rep..

[13] Tanaka H., Hirakawa H., Kosugi S., Nakayama S., Ono A., Watanabe A., Hashiguchi M., Gondo T., Ishigaki G., Muguerza M. (2016). Sequencing and Comparative Analyses of the Genomes of Zoysiagrasses. DNA Res..

[14] Tsuchiya T., Ohta H., Okawa K., Iwamatsu A., Shimada H., Masuda T., Takamiya K.I. (1999). Cloning of Chlorophyllase, the Key Enzyme in Chlorophyll Degradation: Finding of a Lipase Motif and the Induction by Methyl Jasmonate. Proc. Natl. Acad. Sci. USA.

[15] Araya-Garay J.M., Feijoo-Siota L., Veiga-Crespo P., Sánchez-Pérez A., Villa T.G. (2014). Cloning and Functional Expression of Z-Carotene Desaturase, A Novel Carotenoid Biosynthesis Gene from *Ficus carica*. Int. J. Microbiol. Adv. Immunol..

[16] Leng P., Zhang G.L., Li X.X., Wang L.H., Zheng Z.M. (2009). Cloning of *9-Cis-Epoxycarotenoid Dioxygenase* (*NCED*) Gene Encoding a Key Enzyme during Abscisic Acid (ABA) Biosynthesis and ABA-Regulated Ethylene Production in Detached Young Persimmon Calyx. Sci. Bull..

[17] Moummou H., Kallberg Y., Tonfack L.B., Persson B., Rest B.V.D. (2012). The Plant Short-Chain Dehydrogenase (SDR) Superfamily: Genome-Wide Inventory and Diversification Patterns. BMC Plant Biol..

[18] Filling C. (2002). Critical Residues for Structure and Catalysis in Short-Chain Dehydrogenases/Reductases. J. Biol. Chem..

[19] Kavanagh K.L., Jörnvall H., Persson B., Oppermann U. (2008). Medium- and short-chain dehydrogenase/reductase gene and protein families: The SDR superfamily: Functional and structural diversity within a family of metabolic and regulatory enzymes. Cell. Mol. Life Sci..

[20] Jörnvall H., Persson B., Krook M., Atrian S., Gonzalez-Duarte R., Jeffery J., Ghosh D. (1995). Short-Chain Dehydrogenases/Reductases (SDRs). Eur. J. Biochem..

[21] Shimada Y., Wu G.-J., Watanabe A. (1998). A Protein Encoded by *Dinl*, a Dark-Inducible and Senescence-Associated Gene of Radish, Can Be Imported by Isolated Chloroplasts and Has Sequence Similarity to Sulfide Dehydrogenase and Other Small Stress Proteins. Plant Cell Physiol..

[22] Chen Y., Fu X., Mei X., Zhou Y., Cheng S., Zeng L., Dong F., Yang Z. (2017). Proteolysis of Chloroplast Proteins Is Responsible for Accumulation of Free Amino Acids in Dark-Treated Tea (*Camellia Sinensis*) Leaves. J. Proteom..

[23] Liu H. (2022). The Rice Aspartyl-TRNA Synthetase YLC3 Regulates Amino Acid Homeostasis and Chloroplast Development under Low Temperature. Front. Plant Sci..

[24] Soto D., Córdoba J.P., Villarreal F., Bartoli C., Schmitz J., Maurino V.G., Braun H.P., Pagnussat G.C., Zabaleta E. (2015). Functional Characterization of Mutants Affected in the Carbonic Anhydrase Domain of the Respiratory ComplexI in Arabidopsis Thaliana. Plant J..

[25] Guyer L., Hofstetter S.S., Christ B., Lira B.S., Rossi M., Hörtensteiner S. (2014). Different Mechanisms Are Responsible for Chlorophyll Dephytylation during Fruit Ripening and Leaf Senescence in Tomato. Plant Physiol..

[26] Schelbert S., Aubry S., Burla B., Agne B., Kessler F., Krupinska K., Hortensteiner S. (2009). Pheophytin Pheophorbide Hydrolase (Pheophytinase) Is Involved in Chlorophyll Breakdown during Leaf Senescence in *Arabidopsis*. Plant Cell.

[27] Guyer L., Salinger K., Krügel U., Hrtensteiner S. (2017). Catalytic and Structural Properties of Pheophytinase, the Phytol Esterase Involved in Chlorophyll Breakdown. J. Exp. Bot..

[28] Sakuraba Y., Kim D., Kim Y.S., Hörtensteiner S., Paek N.C. (2014). Arabidopsis *STAYGREEN-LIKE* (*SGRL*) Promotes Abiotic Stress-Induced Leaf Yellowing during Vegetative Growth. FEBS Lett..

[29] Clough S.J., Bent A.F. (2010). Floral Dip: A Simplified Method for Agrobacterium-Mediated Transformation of *Arabidopsis thaliana*. Plant J..

[30] Luo H., Hu Q., Nelson K., Longo C., Kausch A.P., Chandlee J.M., Wip J.K., Fricker C.R. (2004). Agrobacterium Tumefaciens-Mediated Creeping Bentgrass (*Agrostis stolonifera* L.) Transformation Using Phosphinothricin Selection Results in a High Frequency of Single-Copy Transgene Integration. Plant Cell Rep..

[31] Kokkirala V.R., Peng Y., Abbagani S., Zhu Z., Umate P. (2010). Subcellular Localization of Proteins of *Oryza sativa* L. in the Model Tobacco and Tomato Plants. Plant Signal. Behav..

[32] Fan Z.-Q., Chen J.-Y., Kuang J.-F., Lu W.-J., Shan W. (2017). The Banana Fruit SINA Ubiquitin Ligase *MaSINA1* Regulates the Stability of *MaICE1* to Be Negatively Involved in Cold Stress Response. Front. Plant Sci..

[33] Ding F., Wang R. (2018). Amelioration of Postharvest Chilling Stress by Trehalose in Pepper. Sci. Hortic..

[34] Bajji M., Kinet J.M., Lutts S. (2002). The Use of the Electrolyte Leakage Method for Assessing Cell Membrane Stability as a Water Stress Tolerance Test in Durum Wheat. Plant Growth Regul..

[35] Winey M., Meehl J.B., O’Toole E.T., Jr G.T. (1978). Conventional Transmission Electron Microscopy. Ultramicroscopy.

[36] Wang G.L., Xiong F., Que F., Xu Z.S., Wang F., Xiong A.S. (2015). Morphological characteristics, anatomical structure, and gene expression: Novel insights into gibberellin biosynthesis and perception during carrot growth and development. Hortic. Res..

[37] Hu B., Jin J., Guo A.-Y., Zhang H., Luo J., Gao G. (2015). GSDS 2.0: An Upgraded Gene Feature Visualization Server. Bioinformatics.

[38] Lescot M. (2002). PlantCARE, a Database of Plant Cis-Acting Regulatory Elements and a Portal to Tools for In Silico Analysis of Promoter Sequences. Nucleic. Acids. Res..

[39] Sudhir K., Glen S., Li M., Christina K., Koichiro T. (2018). MEGA X: Molecular Evolutionary Genetics Analysis across Computing Platforms. Mol. Biol. Evol..

[40] Bailey T.L., Elkan C. (1994). Fitting a mixture model by expectation maximization to discover motifs in biopolymers. Proc. Int. Conf. Intell. Syst. Mol. Biol..

[41] Chen C., Chen H., Zhang Y., Thomas H.R., Xia R. (2020). TBtools: An Integrative Toolkit Developed for Interactive Analyses of Big Biological Data. Mol. Plant.

[42] Yumeng Y., Di Z., Pei Z., Botong L., Sheng-You H. (2017). HDOCK: A Web Server for Protein-Protein and Protein-DNA/RNA Docking Based on a Hybrid Strategy. Nucleic. Acids. Res..

[43] Dong D., Zhao Y., Teng K., Tan P., Liu Z., Yang Z., Han L., Chao Y. (2022). Expression of *ZjPSY*, a Phytoene Synthase Gene from *Zoysia japonica* Affects Plant Height and Photosynthetic Pigment Contents. Plants.

[44] Ma X., Zhang J., Burgess P., Rossi S., Huang B. (2018). Interactive Effects of Melatonin and Cytokinin on Alleviating Drought-Induced Leaf Senescence in Creeping Bentgrass (*Agrostis stolonifera*). Environ. Exp. Bot..

[45] Schmittgen T.D. (2008). Analyzing Real-Time PCR Data by the Comparative CT Method. Nat. Protoc..

[46] Li Y., Zhou C., Yan X., Zhang J., Xu J. (2016). Simultaneous Analysis of Ten Phytohormones in Sargassum Horneri by High-performance Liquid Chromatography with Electrospray Ionization Tandem Mass Spectrometry. J. Sep. Sci..

[47] Imura J., Antoniadi I., Iroká J., Tarkowská D., Strnad M., Ljung K., Novák O. (2018). Plant Hormonomics: Multiple Phytohormone Profiling by Targeted Metabolomics 1. Plant Physiol..

[48] Dong D., Wang M., Li Y., Liu Z., Han L. (2021). Melatonin Influences the Early Growth Stage in *Zoysia japonica* Steud. by Regulating Plant Oxidation and Genes of Hormones. Sci. Rep..

