# Supplementary material for: Expression of a Chlorophyll b Reductase Gene from Zoysia japonica Causes Changes in Leaf Color and Chlorophyll Morphology in Agrostis stolonifera
Source: Int J Mol Sci. 2022 May 27;23(11):6032. doi: 10.3390/ijms23116032 (PMC9181577; doi:10.3390/ijms23116032)
Supplement: Supplementary file 1 [file ijms-23-06032-s001.zip › ijms-1713878-supplementary.pdf]

Expression of a Chlorophyll *b* Reductase Gene from *Zoysia japonica* Causes Changes in Leaf Color and Chlorophyll Morphology in *Agrostis stolonifera*

Di Dong [†], Zhuoxiong Yang [†], Yuan Ma, Shuwen Li, Mengdi Wang, Yinruizhi Li, Zhuocheng Liu, Liebao Han * and Yuehui Chao *

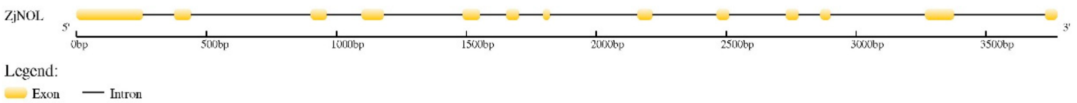

Supplementary Figure S1. Intron/exon arrangement of *ZjNOL*. Exons and introns are depicted as yellow boxes and black lines, respectively.

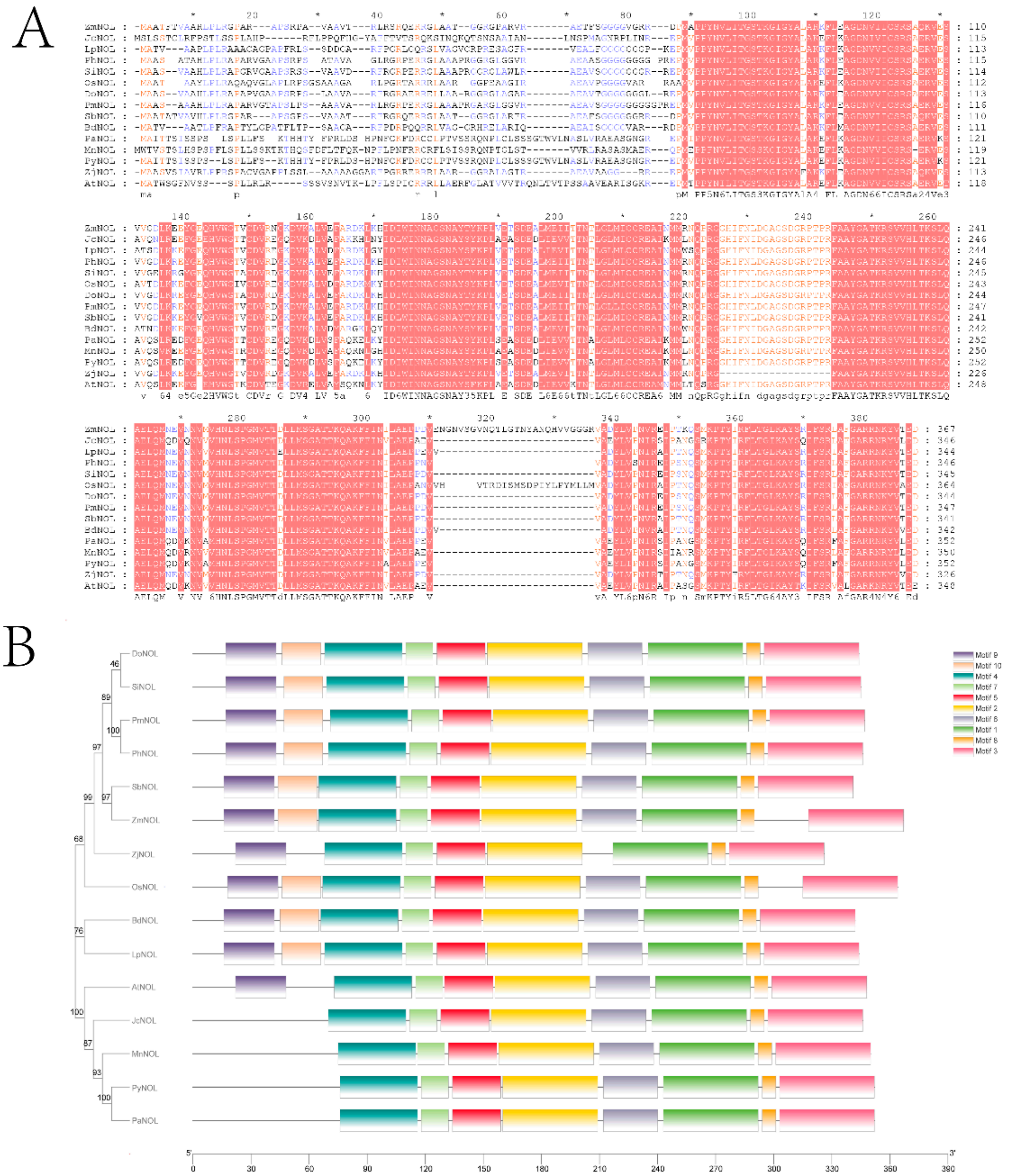

Supplementary Figure S2. Sequence alignment. (A) phylogenetic tree and conserved structure analysis (B) among 15 NOL proteins. Protein sequence accession numbers are as follows: OsNOL, XP_015628273.1, *Oryza sativa*; DoNOL, OEL34851.1, *Dichanthelium oligosanthes*, SbNOL, XP_021306305.1, *Sorghum bicolor*; PmNOL, RLN40231.1, *Panicum miliaceum*; SiNOL, XP_012704137.1, *Setaria italica*; PhNOL, XP_025797592.1, *Panicum hallii*; ZmNOL, PWZ24759.1, *Zea mays*; BdNOL, XP_010231930.1, *Brachypodium distachyon*; JcNOL, XP_012070563.1, *Jatropha curcas*; MnNOL, XP_024026259.1, *Morus notabilis*; PyNOL, PQP93509.1, *Prunus yedoensis*; PaNOL, XP_021829320.1, *Prunus avium*; LpNOL, AQM55941.1, *Lolium perenne*; AtNOL, AT5G04900.1, *Arabidopsis thaliana*.

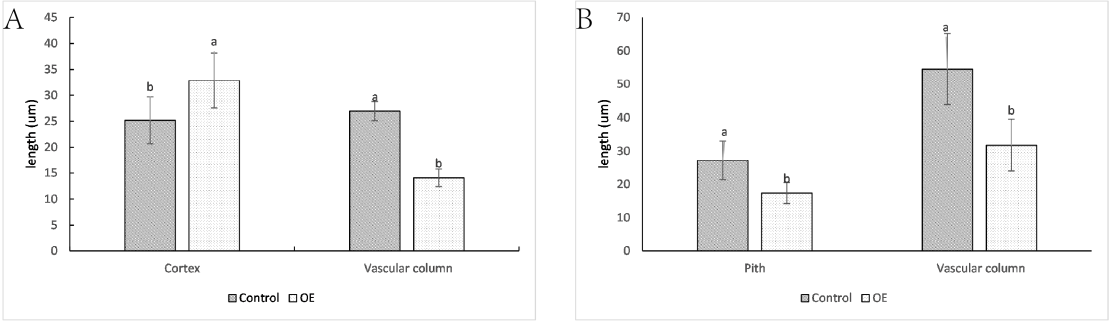

Supplementary Figure S3. Length of cells in leaves and stems of control and OE. A: The cell length of cortex and vascular column cells of leaves in control and OE. B: The cell length of pith and vascular column cells of leaves in control and OE. Different letters above the columns indicate significant differences ($p<0.05$, $n = 20$).

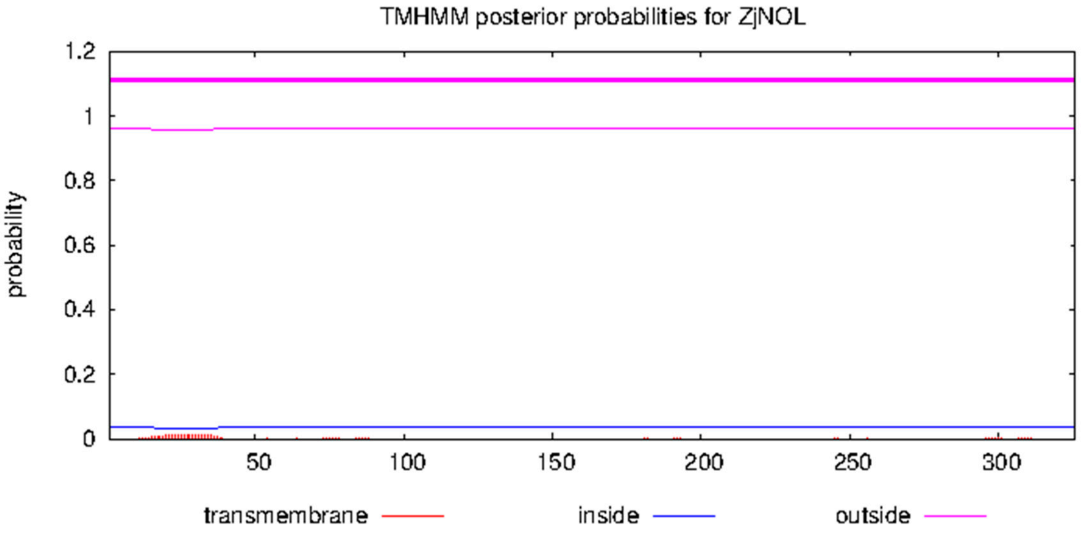

Supplementary Figure S4. Prediction of transmembrane helices in ZjNOL. The prediction results showed that ZjNOL protein contains no transmembrane domain.

Supplementary Table S1. Ten DEGs detected by RNA-Seq and qRT-PCR

Gene ID	Gene name	Log_2ratio	
		RNA-seq	qRT-PCR
c144913.graph_c0	Fructan exohydrolase	1.812982	3.372398
c158384.graph_c1	Trehalose-6-phosphate synthase	1.092785	1.855146
c162235.graph_c0	CK1	1.094827299	2.584788
c130919.graph_c0	YLS3	1.159874	1.551542
c130658.graph_c0	Jacalin-related lectin 9	1.574661	0.855043
c150749.graph_c0	WRKY23	-1.79098	-0.56614
c162887.graph_c0	Lipoxygenase	-1.0241	-0.52376
c154787.graph_c0	Ethylene-responsive transcription factor 9	-1.28581	-1.63941
c162448.graph_c0	Fatty acyl-CoA reductase 1	-1.31387	-0.90685
c143178.graph_c0	Ethylene-responsive transcription factor 4	-1.33728	-0.73319

A total of ten genes including FEH, TPS, CK1, YLS3, JRL9, WRKY23, LOX, ERF9, FAR1, ERF4 were selected for quantitative RT-PCR assays.

Supplementary Table S2. Primers used in the study

Primer name	Primer sequence (5′–3′)
ZjNOL-qPCR-F2	ACGAAGCGAAGTGTTGTG
ZjNOL-qPCR-R2	GGATTGATTAGTAGGGATTGT
ZjNOL-F	CCACCTCGTTTCTTCCTTATTT
ZjNOL-R	CGGTATCCCTTTGTTTGGTG
pGBKT7-ZjNOL-F	AGGAGGACCTGCATATGGCCATGGCTGCCAGCGTCAGCATCGCC
pGBKT7-ZjNOL-R	ATCCCCGGGAATTCGGCCTCATCTTCAACAACATACTTAT
pGAKD7-ZjNYC-F	AGGCCAGTGAATTCCACCCGATGGCCGCCGCCGCCGTCGC
pGAKD7-ZjNYC-R	ATCCCGTATCGATGCCCACCTGTGCCTGGAAGAGGACCAC
3302Y-ZjNOL-F	CTAGTCCTAGGGACGTCAATGGCTGCCAGCGTCAGCATCGCC
3302Y-ZjNOL-R	CTCACCATACGCGTTACAGAATCTTCAACAACATACTTAT
3302YUBI-ZjNOL-F	GTGTTACTTCTGCAGAGGATGGCTGCCAGCGTCAGCAT
3302YUBI-ZjNOL-R	TAATCCAGATCTACCATAGGCTAATCTTCAACAACATACT
YNE-ZjNYC-F	TCGATAGTACTGTCGACCTCATGGCCGCCGCCGCCGTCGC
YNE-ZjNYC-R	ATCCCGGGAGCGGTACCCTCTGTGCCTGGAAGAGGACCAC
YCE-ZjNOL-F	CCTGGCGCGCCACTAGTGGAATGGCTGCCAGCGTCAGCAT
YCE-ZjNOL-R	TCGACAGTACTATCGATGGAATCTTCAACAACATACTTAT
AsCLH1-RT-F	AGGAGGAGGGCTCGTAGGT
AsCLH1-RT-R	GGTCTTCCGTCTGTGCTT
AsNYC-RT-F	GATGGGCTATGTTGACTCTT
AsNYC-RT-R	CTCCACCTGATAACTGAAAT
AsNCED-RT-F	CGGAGACAGACGAGGTTGTAG
AsNCED-RT-R	TGAGACTTTAGGCCACGGTTC
AsZDS-RT-F	ATGGTCCAGCGTGGTAAAGA
AsZDS-RT-R	GGATGGCTAACAGCTCCTCT
ZjACT-F	GGTCCTCTTCCAGCCATCCTTC
ZjACT-R	GTGCAAGGGCAGTGATCTCCTTG
AsACT-F	GAGGTCCTTCCTGATATCCA
AsACT-R	CCTTTTCCAGCCATCTTTCA
AsFEH-F	AGGGAAGGATAGTCCAAGAG
AsFEH-R	TTATAGGCAAGGAATGAGGG
AsTPS-F	CCCTTGTTTACACCCTGTGG
AsTPS-R	GGAGCCTGTGATGAAGACCTA
AsCK1-F	CTGACGACCTTGGGAATAGA
AsCK1-R	TGAACTGTACCACGGAAACG
AsYLS3-F	CACCAACATCTCCGACTGCC
AsYLS3-R	CCCACTCCCAACCACCTCAT
AsJRL9-F	GAGTCCCTCCTTTATCTGCG
AsJRL9-R	ACGGTGGCTGGAAACTACAA
AsWRKY23-F	ACGACCCATTCTCTCCATAGCTCCT
AsWRKY23-R	AGGAGCTATGGAGAGAATGGGTCGT
AsLOX-F	GCGTCTGCCTTGCGAACTCC
AsLOX-R	CGCCGCTCCTAAACCACTCA
AsERF9-F	GGCTGGAGGCTTGGAGGAGA
AsERF9-R	CACCGCCCACCAGAAGAACC
AsFAR1-F	TCGTCATTATTCGTCCCACC
AsFAR1-R	CTTGCCCTTTCTCATTCCAG
AsERF4-F	TCCTCGGGTCCAGGAACAGG
AsERF4-R	CCAACTTCCCTCCCACCTCC

Supplementary Table S3. Protein sequence of 15 NOL proteins

	Protein sequence
ZjNOL	MAASVSIAVRLPPRSPACVGAPPLSSLAAAAAGGARFPGRRERRRLAARG<br>GRALAGIRAEAVAAGGRREPMVPPYNVLITGSTKGIGYAFAKKFLEAGD<br>NVIICSRSAQKVESVVGDLKKEYGEQHVWGTVCDVRDGKDVKALVEFA<br>RDKLKHIDIWINNAGSNAYTYKPLVETSDEALMEVITTNTLGLMICCREAI<br>NMMRNQPRGFAAYGATKRSVVHLTKSLQAELQMNEVNNVMVHNLSP<br>GMVTTDLLMSGATTKQAKFFINILAEPPDVVADYLVPNIRTIPTNQSMKP<br>TYFRYLTGLKAYSRIFSRLAFGARRNKYVVED
OsNOL	MAATAAYLPLRAQAQVGLAPLRPSGSAAAGARLPGRTARRRLAARGGP<br>EAAGIRAEAVPGGGGVARRAAMVPPYNVLITGSTKGIGYALAKEFLKAG<br>DNVVICSRSAERVESAVTDLKKEFGEQHVWGIVCDVREGKDVKALVDFA<br>RDKMKYIDIWINNAGSNAYSYKPLVETSDEALMEVITTNTLGLMICCREA<br>INMMRNQPRGGHIFNIDGAGSDGRPTPRFAAYGATKRSVVHLTKSLQAE<br>LQMNEVNNVMVHNLSPGMVTTDLLMSGATTKQAKFFINILAEPANVV<br>HVTRDISMSDPIYLPYMLLMVADYLVPNIRAIPTNQSMKPTYIRFLTGLKA<br>YSRIFSRIAFGARRNKYVAED
DoNOL	MAASVAAHLPLRAPAPVGAAPSRPSLAAVARFRGRAERRELLAARGGRG<br>LAGARAEAVTGGGGGGGLREPMVPPYNVLITGSTKGIGYALARKFLEAG<br>DNVVICSRSAEKVESVVGDLKREYGEQHVWGTACDVRDGKDVKALVEF<br>ARDKLKHIDIWINNAGSNAYTYKPLVETSDEALMEIITTNTLGLMICCRE<br>AINMMRNQPRGGHIFNLDGAGSDGRPTPRFAAYGATKRSVVHLTKSLQ<br>AELQMNEVNNVMVHNLSPGMVTTDLLMSGATTKQAKFFINILAEPPDV<br>VADYLVPNIREIPSNQSMKPTYIRFLTGLKAYSRIFSRLAFGARRNKYVTED
SbNOL	MAATATVAVHLPLRGPARAPSGPSVAAATRFRGRQERRGLAATGGRGLA<br>RFRAEAFSGGGGGGRRDPMVPPYNVLITGSTKGIGYALARKFLEAGDNVI<br>ICSRSAQKVESVVGDLKKEYGVQHVWGTVCDVRDGKDVKALVEFARDK<br>LKHIDLWINNAGSNAYTYKPLVETSDEALMEIITTNTLGLMICCREAINM<br>MRNQPRGGHIFNLDGAGSDGRPTPRFAAYGATKRSVVHLTKSLQAELQ<br>MNEVNNVMVHNLSPGMVTTDLLMSGATTKQAKFFINILAEPPDVVADY<br>LVPNVRAIPTNQSMKPTYIRFLTGLKAYSRIFSRLAFGARRNKYVTED
PmNO<br>L	MAASAAAHLPLRAPARVGTAPSLPSAAAVARLRGRPERRGLAAAPRGA<br>RGLGGVRAEAVSGGGGGGGGPREPMVPPYNVLITGSTKGIGYALARKF<br>LEAGDNVVICSRSAEKVESVVGDLKREYGEQHVWGTVCDVRDGKDVKA<br>LVEFARDKLKHIDIWINNAGSNAYTYKPLVETSDEALMEIITTNTLGLLICC<br>REAINMMRNQPRGGHIFNLDGAGSDGRPTPRFAAYGATKRSVVHLTKSL<br>QAELQMNEVNNVMVHNLSPGMVTTDLLMSGATTKQAKFFINILAEPPD<br>VVADYLVPNIREIPSNQSMKPTYIRFLTGLKAYSRIFSRLAFGARRNKYVTE<br>D
SiNOL	MAASVAAHLPLRAPGRVGAAPSRSSVAAVDRFRGRPERRGLAAAPRGG<br>RGLAWLRAEAVSGGGGGGGRREPMVPPYNVLITGSTKGIGYALARKFLE<br>AGDNVIICSRSAEKVESVVGELKRGYGEQHVWGTACDVRDGKDVKALV<br>EFARDKLKHIDIWINNAGSNAYTYKPLVETSDEALMEIITTNTLGLMICCR<br>EAINMMRNQPRGGHIFNLDGAGSDGRPTPRFAAYGATKRSVVHLTKSLQ

	AELQMNEVNNVMVHNLSPGMVTTDLLMSGATTKQAKFFINILAEPPDV
	VADYLVPNIREIPSNQSMKPTYIRFLTGLKAYSRIFSRLAFGARRNKYVTED
PhNOL	MAASATAHLPLRAPARVGAAPSRPSATAVAGLRGRPERRGLAAAPRGGR
	GLGGVRAEAASGGGGGGGGPREPMVPPYNVLITGSTKGIGYALARKFLE
	AGDNVVICSRSAEKVESVVGDLKREYGEQHVWGTVCDVRDGKDVKALV
	EFARDKLKHIDIWINNAGSNAYTYKPLVETSDEALMEIITTNTLGLMICCR
	EAINMMRNQPRGGHIFNLDGAGSDGRPTPRFAAYGATKRSVVHLTKSLQ
	AELQMNEVNNVMVHNLSPGMVTTDLLMSGATTKQAKFFINILAEPPNV
	VADYLVSNIREIPSNQSMKPTYIRFLTGLKAYSRIFSRLAFGARRNKYVTED
ZmNO	MAATFTVAARLPLRGPARAPSRPAVAAVTRLRSRQERRGLAATGGRGPA
L	RVRAETFSGGGGVGRRDPMAPPYNVLITGSTKGIGYALARKFLEAGDNVI
	ICSRSAQKVESVVGDLKEEYGEQHVWGTVCDVRNGKDVKALVEFARDK
	LKHIDIWINNAGSNAYTYKPLVETSDEALMEIITTNTLGLMICCREAINM
	MRNQPRGGHIFNLDGAGSDGRPTPRFAAYGATKRSVVHLTKSLQAELQ
	MNEVNNVMVHNLSPGMVTTDLLMSGATTKQAKFFINILAEPPDVENGN
	VSGVNQTLGTNYANQHVVGGGRVADYLVPNVREIPTKQSMKPTYIRFLT
	GLKAYSRIFSRLAFGARRNKYVTED
BdNOL	MATVAATLPFRAPTYLGPATFLTPSAAGARFPDRPQQRRLVAGGRHRELA
	RIQAEAISGGGGVARRDPMVPPYNVLITGSTKGIGYALAKKFLMAGDNVI
	ICSRSAERVESATNDLKKEFGEQHVWGTVCDVREGKDVKALVDYARGKL
	QYIDIWINNAGSNAYSYKPLVETSDEALMEVITTNTLGLMICCREAINMM
	WNQPRGGHIFNIDGAGSDGRPTPRFAAYGATKRSVVHLTKSLQAELQM
	NEVNNVVVHNLSPGMVTTDLLMSGATTKQAKFFINILAEPPDVVADYLV
	PNVRAIPTNQSMKPTYIRFLTGLKAYSRIFSRLAFGARRNKYVVED
JcNOL	MSLSSTCLRFPSTLSSPLAHPRFLPPQFHGYAFFTVTSRQKSINQKQTSNSA
	AIANLNSPMAGNRPLINREPMVPPYNVLITGSTKGIGYALAKEFLKAGD
	NIVICSRSAERVESAVQNLREEFGEEHVWGTTCDVREGQDVKDLVAFAK
	KHLNYIDLWINNAGSNAYSYKPLAEASDEDLIEVVTTNTLGLMICCREAI
	KMMLNQPRGGHIFNIDGAGSDGRPTPRFAAYGATKRSVVHLTKSLQAEL
	QMQDVQNVVVHNLSPGMVTTDLLMSGATTKQAKFFINVLAEPAEVVAE
	YLVPNIRSIPANGSRKPTYIRFLTGIKAYSQIFSRLAFGARRNRYVLED
MnNO	MWTVSTSLHSPSPFLSPLLSSKTKTHQSFDFLTFQKNPFLPNFRRCRFLSISS
L	RQNPTGLSTVVRLRASASMAERQPMEPPFNVLITGSSKGIGYALAKEFLK
	AGDNVVICSRSERVESAVQSVKEEFGEQHVWGTRCDVREGQDVKDLVAF
	AQRNLGHIDIWINNAGSNAYSYKPLAEASDEDLIEVVTTNTLGLMICCRE
	AIKMMLNQPRGGHIFNIDGAGSDGRPTPRFAAYGATKRSVVHLTKSLQA
	ELQMQDVRNVVVHNLSPGMVTTDLLMSGATTKQAKFFINVLAEPAEVV
	AEYLVPNIRSIIANRSMKPTYIRFLTGLKAYSQIFSRLAFGARRNRYLLED
PyNOL	MAITTSISSPSLSPLLFSKTHHTYFPRLDSHPNFCKFDRCCLPTVSSRQNPL
	CLSSSGTWVLNASLVRAEASGNGREPMVPPYNVLITGSTKGIGYALAKEF
	LKAGDNVIICSRSAERVKSAVQSLREDFGEQHVWGTTCDVREGQDVKDL
	VSFAQKELKYIDIWINNAGSNAYSYKPLSEASDEDLIEVVTTNALGLMLC
	CREAIKMMLNQPRGGHIFNIDGAGSDGRPTPRFAAYGATKRSVVHLTKS
	LQAELQMQDVKNVAMHNLSPGMVTTDLLMSGATTKQAKFFINALAEP

	PEVVAEYLIPNIRSIPANGSMKPTYIRFLTGIKAYSQIFSRFAFGARRNRYVL<br>ED
PaNOL	MAITTSISSPSLSPLLFSKTHHTYFPRLDSHPNFCKFDRCCLPTVSSRQNPL<br>CLSSSGTWVLNASLVRAEASGNGREPMIPPYNVLITGSTKGIGYALAKEFL<br>KAGDNVIICSRSAERVKSAVQSLREDFGEQHVWGTTCDVREGQDVKDLV<br>SFAQKELKYIDIWINNAGSNAYSYKPLSEASDEDLIEVVTTNALGLMLCC<br>REAIKMMLNQPRGGHIFNIDGAGSDGRPTPRFAAYGATKRSVVHLTKSL<br>QAELQMQDVKNVAMHNLSPGMVTTDLLMSGATTKQAKFFINVLAEPPE<br>VVAEYLVPNIRSIPANGSMKPTYIRFLTGIKAYSQIFSRFAFGARRNRYVLE<br>D
LpNOL	MATVAAPLPLRAAACAGPAPFRLSSDDGARFPGRLGQRSLVAGVCRPRE<br>SAGFRVEALFGGGGGGGPKEPMVPPYNVLITGSTKGIGYALAKKFLKAG<br>DNVVICSRSAERVESATSDLKKEFGEQHVWGTVCDVREGKDVKALVDFA<br>RDKLGYIDIWINNAGSNAYSFKPLVETSDEALIEVITTNTLGLMLCCREAI<br>NMMWSQPRGGHIFNIDGAGSDGRPTPRFAAYGATKRSVVHLTKSLQAEL<br>QMNEVNNVVVHNLSPGMVTTELLMSGATTKQAKFFINILAEPPEVVAD<br>YLVPNIRAIPTNQSMKPTYIRFLTGLRAYSRIFSRIAFGARRNKYVTED
AtNOL	MATWSGFNVSSSPLLRLRSSSVSNVTKLPFLSPICRRRLLAERFGLATVVVT<br>RQNLTVTPSSAAVEARISGKREPMTPPYNILITGSTKGIGYALAREFLKAG<br>DNVVICSRSAERVETAVQSLKEEFGEHVWGTKCDVTEGKDVRELVAYSQK<br>NLKYIDIWINNAGSNAYSFKPLAEASDEDLIEVVKTNTLGLMLCCREAM<br>NMMLTQSRGGHIFNIDGAGSDGRPTPRFAAYGATKRSVVHLTKSLQAEL<br>QMQDVKNVVVHNLSPGMVTTDLLMSGATTKQAKFFINVLAEPAEVVAE<br>YLVPNIRAIPASGSMKPTYIRFLTGIKAYTKIFSRVALGARKNRYVTEE
